# PHD finger protein 20-like protein 1 (PHF20L1) in ovarian cancer: from its overexpression in tissue to its upregulation by the ascites microenvironment

**DOI:** 10.1186/s12935-021-02425-6

**Published:** 2022-01-06

**Authors:** Dulce Rosario Alberto-Aguilar, Verónica Ivonne Hernández-Ramírez, Juan Carlos Osorio-Trujillo, Dolores Gallardo-Rincón, Alfredo Toledo-Leyva, Patricia Talamás-Rohana

**Affiliations:** 1 Centro de Investigación y de Estudios Avanzados del Instituto Politécnico Nacional, Av. Instituto Politécnico Nacional 2508, Col. San Pedro Zacatenco, Delegación Gustavo A. Madero, 07360 Mexico City, Mexico; 2 Instituto Nacional de Cancerología, Av. San Fernando No. 22, Col. Sección XVI, Delegación Tlalpan, 07360 Mexico City, Mexico

**Keywords:** Ascites, Immunohistochemistry, Oncogene, Ovarian cancer, PHF20L1

## Abstract

**Background:**

Ovarian cancer is the most aggressive gynecological malignancy. Transcriptional regulators impact the tumor phenotype and, consequently, clinical progression and response to therapy. PHD finger protein 20-like protein 1 (PHF20L1) is a transcriptional regulator with several isoforms, and studies on its role in ovarian cancer are limited. We previously reported that PHF20L1 is expressed as a fucosylated protein in SKOV-3 cells stimulated with ascites from patients with ovarian cancer.

**Methods:**

We decided to analyze the expression of PHF20L1 in ovarian cancer tissues, determine whether a correlation exists between PHF20L1 expression and patient clinical data, and analyze whether ascites can modulate the different isoforms of this protein. Ovarian cancer biopsies from 29 different patients were analyzed by immunohistochemistry, and the expression of the isoforms in ovarian cancer cells with or without exposure to the tumor microenvironment, i.e., the ascitic fluid, was determined by western blotting assays.

**Results:**

Immunohistochemical results suggest that PHF20L1 exhibits increased expression in sections of tumor tissues from patients with ovarian cancer and that higher PHF20L1 expression correlates with shorter progression-free survival and shorter overall survival. Furthermore, western blotting assays determined that protein isoforms are differentially regulated in SKOV-3 cells in response to stimulation with ascites from patients with epithelial ovarian cancer.

**Conclusion:**

The results suggest that PHF20L1 could play a relevant role in ovarian cancer given that higher PHF20L1 protein expression is associated with lower overall patient survival.

**Supplementary Information:**

The online version contains supplementary material available at 10.1186/s12935-021-02425-6.

## Background

Among all the gynecological cancers, ovarian cancer is considered the cancer with the worst prognosis and the highest mortality rate [1]. Most patients are diagnosed with advanced-stage tumors [2] because the manifestation of nonspecific symptoms makes their detection and diagnosis difficult. The late detection of the disease is directly related to its high mortality rate. The diagnosis of ovarian cancer typically involves Ca125 and transvaginal ultrasound; these methods have some limitations, and the need for more specific and sensitive diagnostic tools remains [3]. New molecules are being studied to determine their relevance for the development of ovarian cancer, improve diagnosis, and treat the disease to reduce patient mortality.

In cancer, there are a wide variety of altered transcriptional regulators that control processes to promote cancer progression and metastasis [4]. Similar to many other cancers, ovarian cancer is closely associated with genetic disorders [5]. Therefore, the set of characteristics that give rise to the tumor phenotype, including those that affect the clinical course and the response to therapy, are controlled by deregulated transcriptional programs that operate on tumor cells [6]. Knowing more about these transcriptional regulators in ovarian cancer could generate useful tools to diagnose, treat, or understand tumor biology.

PHF20L1 (*PHD finger protein 20-like 1*) is a transcriptional regulator that is present at low levels in almost all cell types [7, 8], where it binds to key molecules with which it exerts transcriptional repression [9]. Approximately 21 splice variants are known, of which three are expressed [7]. However, to date, its function has not been clearly described. New fucosylated proteins were previously reported as potential biomarkers in ovarian cancer by our laboratory [10]. In addition to other proteins, PHD finger 20-like protein 1 was found in the list of fucosylated proteins. Studies carried out in breast cancer demonstrated an increase in PHF20L1 expression associated with a poor prognosis. In ovarian cancer, alterations in the gene copy number of PHF20L1 were previously found by another research group [11]. However, the expression of the protein in ovarian cancer has not yet been analyzed [12]. It is also unknown whether the tumor microenvironment, namely, malignant ascites from ovarian cancer patients [13, 14], modulate the expression of this protein. Thus, in the present work, we focus on the relevance of PHF20L1 expression in tumor tissue sections and ovarian cancer cell lines and the correlations between its expression level and the clinical data of ovarian cancer patients. Furthermore, we determined whether ascites could modify PHF20L1 protein expression levels in OVCAR-3 and SKOV-3 cells.

The results showed increased PHF20L1 expression in ovarian cancer tissue compared with healthy ovaries; overexpression showed a negative trend with patient progression-free survival (PFS) and overall survival (OS). When the cell lines were analyzed, we found that the majority of the ascites tested (8/10) stimulated overexpression of the protein. Based on these results, we conclude that PHF20L1 is a protein that can be modulated by ascites components and suggest that its function and expression are important in cancer progression given that its overexpression in tumor tissues was associated with a worse prognosis. However, the conclusions of this work are limited due to the need to analyze a larger number of tissue samples from ovarian cancer patients and to study the association between PHF20L1 expression and the response to treatment, which could provide additional information about the role of the protein in tumor progression.

## Material and methods

### Biological samples

Paraffin-embedded tissues were used for immunohistochemistry assays. In total, 33 slides with paraffin-embedded sections of tumor tissue were employed. Of these slides, 29 corresponded to tumor tissue sections from patients with different histological subtypes of epithelial ovarian cancer (Table 1), whereas two slides corresponded to cervical cancer and breast cancer. These two last slides were used as a comparison to other cancer tissues. Additionally, two negative controls consisting of cancer-free ovarian tissue with normal appearance obtained from adjacent areas to the tumor were included.

**Table 1:** Clinical and pathological characteristics of ovarian cancer patients whose tumor tissue sections were employed in the immunohistochemistry analysis

Characteristics	N = 29
Age (years)	
Mean ± SD	49 ± 10.65
Median (Range)	50 (28–67)
CA 125 (U/mL)	
Mean ± SD	1097.18 ± 1515.6
Median (range)	365 (11.4–5958)
Clinical stage% (n/N)	
IA	6.9 (2/29)
IC	3.45 (1/29)
IIIA	6.9 (2/29)
IIIB	3.45 (1/29)
IIIC	58.6 (17/29)
IVA	13.8 (4/29)
IVB	6.9 (2/29)
Histology% (n/N)	
HGSP	55.17 (16/29)
LGSP	6.9 (2/29)
Endometrioid	20.68 (6/29)
Clear cells	13.8 (4/29)
Mucinous	3.45 (1/29)

Paraffin-embedded tissues were obtained from patients diagnosed with epithelial ovarian cancer (EOC) and cervical and breast cancer, as appropriate, at the Instituto Nacional de Cancerología (INCan) under the approval of the Research and Ethics Committees (012/018/GII) (CB764/12).

Ascites were obtained from patients diagnosed with epithelial ovarian cancer (EOC) at the Instituto Nacional de Cancerología (INCan) under the approval of the Research and Ethics Committees (009/029/GOI) (CB/549/09). All the samples were used based on the principles of the Declaration of Helsinki regarding the ethical principles of medical research involving human samples, and patients signed the corresponding informed consent form. Ascites were collected by medical personnel. Ascites samples contaminated with blood were excluded from the study. Approximately 100 mL was centrifuged at 1000 rpm for 10 min to recover the cell-free supernatant and the cell pellet. Only cell-free ascites (supernatants) were further employed for experiments and were stored at − 70 °C until use. Before use, ascites were defrosted and warmed at room temperature. The clinical and pathological characteristics of the epithelial ovarian cancer patients whose ascites were used in this project are described in Table 2.

**Table 2:** Clinical and pathological characteristics of ovarian cancer patients whose ascites were employed in western blot and immunofluorescence analyses

Characteristics	% (n/N) N = 10
Age (years)	
Mean ± SD	57 ± 9.9
Median (range)	55 (43–73)
CA 125 (U/mL)	
Mean ± SD	2781.71 ± 2760.741
Median (range)	2225.55 (570.5–13,730.2)
Clinical stage	
IIIC	40 (4/10)
IVA	10 (1/10)
IVB	50 (5/10)
Histology	
HGSP	90 (9/10)
Endometrioid	10 (1/10)

The reference cell line SKOV-3 (ATCC HTB-77, purchased in 2010) was used for in vitro experiments. Additionally, OVCAR-3 (ATCC HTB-161, purchased in 2010) cell line was used for comparative purposes in the development of this work. The SKOV-3 model has been used previously in our laboratory as described [15]. Briefly, SKOV-3 cells were cultured in McCoy’s 5A (Corning, 10-050-CVR) culture medium (15 mL) supplemented with 10% fetal bovine serum (Corning, 35-010-CV) and 1% penicillin/streptomycin (PAA, P11-010) at 37 °C and 5% CO_2_. OVCAR-3 cells were grown in RPMI 1640 culture medium (Corning, 10-004-CM) supplemented with 10% fetal bovine serum and bovine insulin (Millipore, I0516). These cultures were grown until cells reached 75% confluence. Then, the medium was discarded before the subsequent addition of ascites (15 mL). Cells maintained in culture medium were used as controls. The cells were maintained under ascites stimulus at 37 °C and 5% CO_2_ for the indicated periods. SKOV-3 and OVCAR-3 cells were maintained under ascites stimulus at 37 °C and 5% CO_2_ for 24 or 48 h.

SKOV-3 cell line was typified in 2019 by STR-type genetic markers amplified at the National Institute for Genomic Medicine (INMEGEN). Experiments to test for the presence of contaminating mycoplasma in cultures are routinely performed.

### Immunohistochemistry

Paraffin-embedded tissues were used to perform PHF20L1 immunohistochemistry. Briefly, tissues were dewaxed overnight at 50 °C. The specimens were hydrated in xylol (3 × 5 min) and decreasing ethanol solutions (100, 95, 90, 85, 80%; 30 s each). Antigenic recovery was carried out using 0.1 N citrate buffer at 121 °C for 20 min. Slides were rinsed 3 × with 1 × phosphate-buffered saline (PBS) for 5 min each. Endogenous peroxidase was blocked by incubating the slide in hydrogen peroxide solution for 1 h at room temperature and washed again with 1 × PBS (5 × 5 min). PBS-10% milk was added for 1 h at room temperature to block nonspecific binding. Then, the primary anti-PHF20L1 antibody (Sigma-Aldrich HPA028417) (1:100 dilution, 1 × PBS-SFB 10%) was added with overnight incubation at 4 °C. The next morning, the slides were rinsed 5 × with 1 × PBS, and the secondary horseradish peroxidase-conjugated anti-rabbit IgG antibody (HRP) was placed in a 1:100 dilution and incubated for 2 h at room temperature. Corresponding washes were performed, and diaminobenzidine substrate (Invitrogen, Catalog number 002020) was added to visualize the reaction followed by incubation for 5 min. Finally, hematoxylin counterstaining was performed, and the slides were fixed for observation under a light microscope (Nikon, E100).

### Immunohistochemistry evaluation of PHF20L1 positivity (immunoreactive score)

The QuPath program (https://github.com/qupath) was used to quantify the number of positive cells and the signal intensity. The determination of the positive signal was assigned by the software, and three levels of signal intensity are indicated: slightly positive (1 +), moderately positive (2 +), and strongly positive (3 +). Areas without staining were classified as negative (Neg).

Additionally, to generate the survival curves, the immunoreactive scores were calculated for each slide [16]. For this analysis, values were assigned to the percentage of positive cells (A) and the intensity of the signal (B); the result of the A value multiplied by the B value, which ranges from 0 to 12, allows us to classify the expression as negative, weak, moderate or intense.

### Treatment of SKOV-3 cells with ovarian cancer ascites and cellular protein extract

After the treatment of SKOV-3 cells (24 or 48 h) with ten different ascites samples and the control condition (culture medium), cell cultures were washed with sterile 1 × PBS and were subsequently recovered using trypsin solution (Corning, 25-051-CI). Cell lysis was carried out using RIPA buffer (5 mM Tris–HCl, 2 mM EDTA, 50 mM NaCl, and 1% Nonidet P-40) with a cocktail of protease and phosphatase inhibitors (1 mg/ml aprotinin and leupeptin and 1 mM PMSF, NaF, and Na_3_VO_4_). Finally, the samples were quantified using a DC Protein Assay Kit (Bio-Rad, 500-0114).

### SDS-PAGE and Western blotting

The total protein extracts (60 µg) were visualized by 10% SDS-PAGE. The gel was transferred to a nitrocellulose membrane. The nitrocellulose membrane was blocked with 5% milk for 1 h at room temperature. Then, the membrane was washed 3× with TBS-Tween 0.1% for 15 min each time and incubated with the antibody against PHF20L1 (Sigma-Aldrich HPA028417) (1:2000 dilution in TBS-T 0.1% with milk 1%) overnight at 4 °C. Then, the membrane was washed 3× with TBS-T (containing 0.1% Tween) and incubated with HRP goat anti-rabbit (IgG) secondary antibody (1:10,000 dilution in 0.1% TBS-T with 1% milk) for 1 h at room temperature. Again, the membrane was washed 3× with TBS-T. Finally, the cells were incubated with Super Signal West Femto (Thermo Fisher Scientific, 34094) for visualization.

As a loading control for western blot to normalize the levels of protein detected, a GAPDH antibody was employed (1:15,000 dilution in 0.1% TBS-T with 1% milk) (Genetex GTX100118).

### Immunofluorescence

SKOV-3 and OVCAR-3 cells adhered to coverslips were subjected to different treatment conditions. Upon completion, the cells were fixed with 4% paraformaldehyde for 1 h at 37 °C. Cells were rinsed 3× with filtered 1× PBS and permeabilized with a 0.2% solution of Triton X-100 in 1× PBS for 15 min at room temperature with stirring. Cells were rinsed thrice with filtered 1× PBS and blocked with 10% FBS for 1 h at 37 °C. The primary antibody (Sigma-Aldrich HPA028417) was added at a 1:100 dilution in 1× PBS and incubated overnight at 4 °C in a humid chamber. On the next day, cells were rinsed 3× with 1× PBS. TRITC goat anti-rabbit (IgG) secondary antibody (ab50598, Abcam) was added to OVCAR-3 cells and incubated for 1 h at 37 °C under protection from light. For the SKOV-3 assays, a different secondary antibody was employed: Alexa Fluor 647 donkey anti-rabbit (IgG) secondary antibody (1:100 dilution) (711-605-152, Jackson InmunoResearch Laboratories), this with the objective to improve the signal of the protein. Vectashield with DAPI (Cat. No. H-1200) was added, and the coverslip was mounted on a slide. The assembled samples were stored at − 20 °C protected from light until observation under a Carl Zeiss LMS 700 confocal microscope. ZEN 2012 software was used to analyze the samples (Carl Zeiss).

### Statistical analysis

Densitometric analysis was performed using ImageJ software. Statistical analyses were performed using GraphPad Prism 7 software. For western blot comparisons, Student’s *t*-test was performed. The Spearman’s correlation test was performed to assess the correlation between PHF20L1 expression and the clinical data of the patients, and the value of ρ was reported in the corresponding tables. Student’s *t*-test comparatively evaluated the control and problem conditions. Comparisons between groups were performed using ANOVA. The average and the corresponding standard deviations of in vitro assays performed in triplicate are included in the graphs. Significant differences are indicated as follows: *ρ < 0.01, **ρ < 0.001, ***ρ < 0.0001.

Patient survival was analyzed using the Kaplan–Meier method. Overall survival (OS), which is defined as the length from time of diagnosis to death or the last follow-up, and progression-free survival (PFS), which is defined as the time length from a random assignment in a clinical trial to a diagnosis of progressive disease or relapse, of the 29 patients with EOC were determined. Survival data of patients alive without progression or those who died due to other diseases were censored.

Expression values (arbitrary units obtained from the densitometric analysis) obtained from western blotting data were used to categorize patients into two groups: low or high expression of any of the isoforms. In each cohort, each patient was further classified as follows:

For the “a” isoform:

Low: value from 0.3 to 0.45.

High: Value from 0.57 to 0.76.

For the "b" isoform:

Low: value from 0.72 to 0.8

High: value from 0.93 to 1.36.

For the "c" isoform:

Low: value from 0.05 to 0.15.

High: value from 0.22 to 0.52.

## Results

### Epithelial ovarian cancer tissue overexpressed PHF20L1

**Fig. 1:**
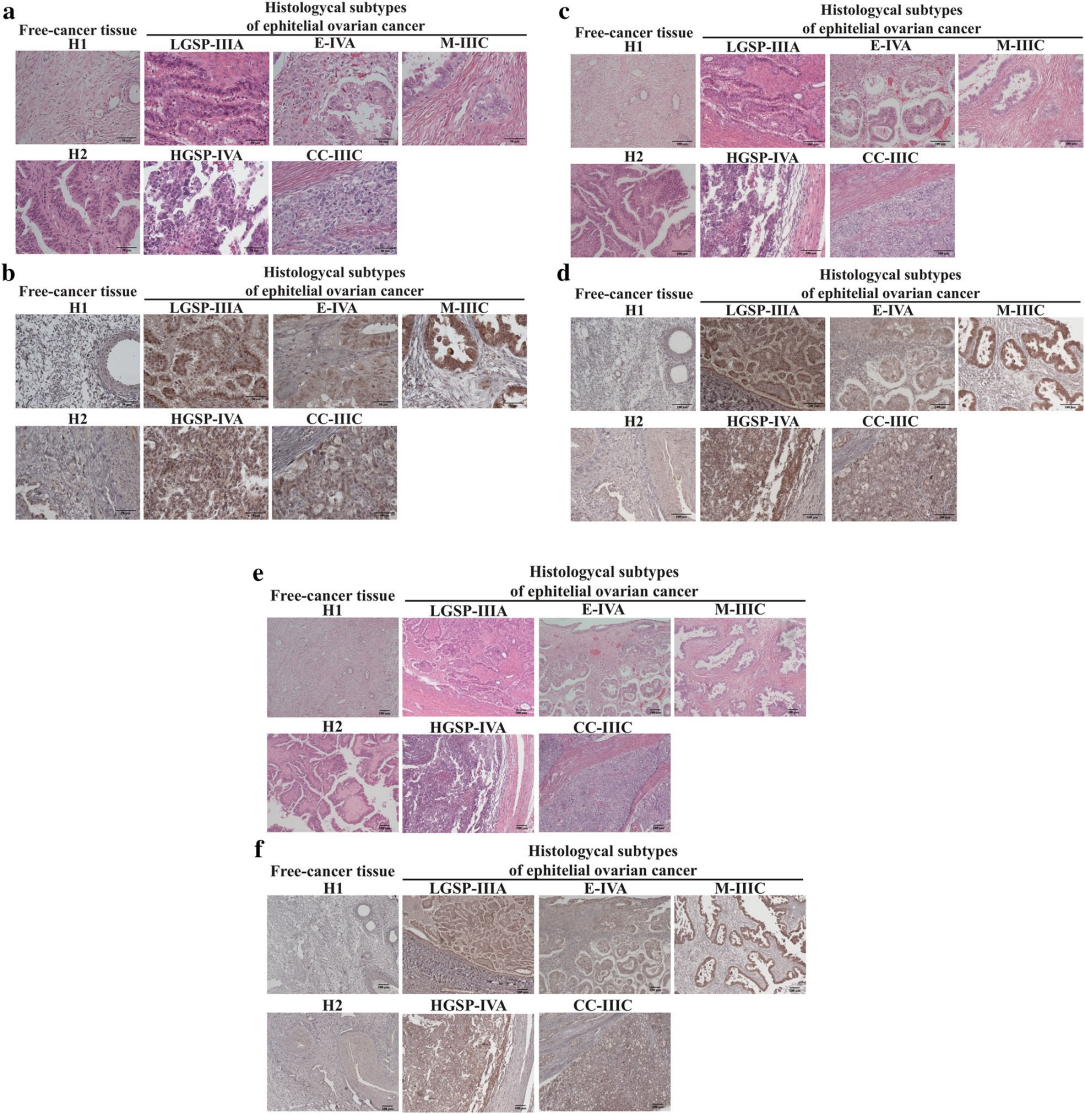
Expression of PHF20L1 in sections of EOC histological subtypes. **a** Hematoxylin and eosin (H&E) staining of control tissues (cancer-free tissues: H1 and H2) and five sections corresponding to five different patients (out of 29 total samples analyzed; see Additional file 1: Fig. S1). In the H&E staining, nuclei appear as purple, and the cytoplasm appears as shades of pink. **b** IHC staining corresponding to control tissue and five sections of tumor tissue. The positive signal in the IHC analysis appears as different shades of brown. LGSP: low-grade serous papillary; HGSP: high-grade serous papillary; E: endometrioid; CC: clear cells; M: mucinous; followed by the clinical stage. The images shown were observed at 40× magnifications (**a** and **b**); 20× magnifications (**c** and **d**); 10× magnifications (**e** and **f**)

PHF20L1 is a protein discreetly expressed in many tissues; high expression is typically present in glandular cells, as reported for the thyroid gland in The Human Protein Atlas https://www.proteinatlas.org/https://www.proteinatlas.org/ [8]. Using immunohistochemistry, 29 different biopsies of patients with different histological subtypes of epithelial ovarian cancer were analyzed (Table 1 and Additional file 6: Table S1). Two biopsies of ovarian cancer-free tissue were employed as controls for "normal" expression (Fig. 1). For comparative purposes, tissue from one cervical and one breast cancer biopsy were included.

**Fig. 2:**
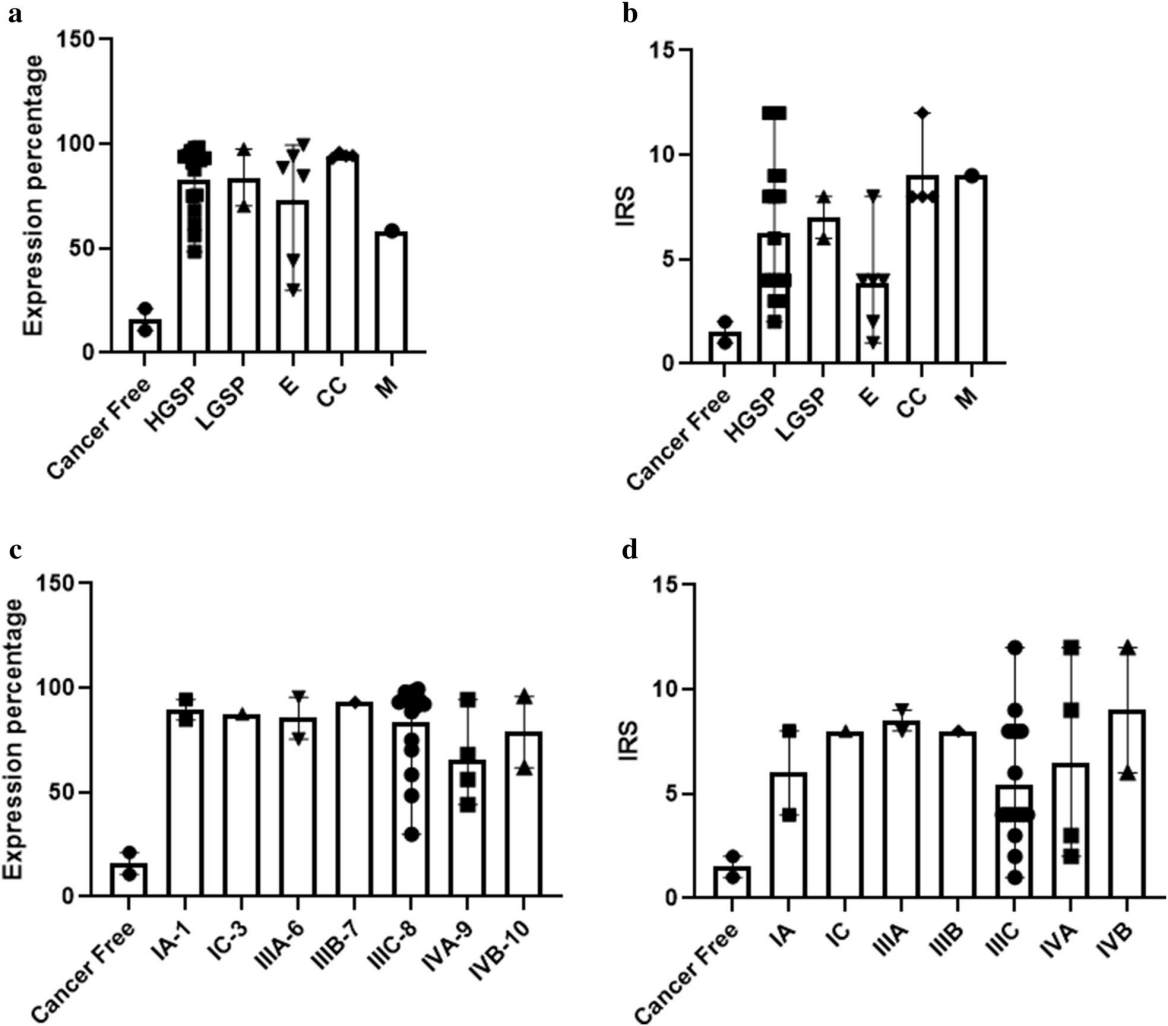
Graphs showing the distribution of PHF20L1 expression levels in biopsies. Samples were grouped by histological subtype (**a**, **b**) or clinical stage (**c**, **d**) in relation to the percentage of expression (**a**, **c**) and immunoreactivity (IRS) values (**b**, **d**), which were reported on the y-axis as Expression (%) or as Immunoreactive Score (ranging from 0 to 12), respectively

Control tissues (H1 and H2; Fig. 1) showed low protein expression in the cells. Breast and cervical cancer tissue showed low expression distributed throughout the tissue section analyzed (Additional file 6: Table S1, Additional file 2: Fig. S2). When epithelial ovarian cancer tissues were analyzed, high protein expression was observed in most tissues, especially in all regions containing cells with high cytoplasmic content and large nuclei (Fig. 1). In some samples, it was possible to observe a positive signal mainly inside the nucleus (Additional file 3: Fig. S3). No differences in the expression level were noted among histological subtypes or clinical stages (Fig. 2). In addition, when the immunoreactive score was calculated, the results showed an increase in signal in tumor tissues form ovarian cancer patients compared to healthy tissue, this signal ranged from low to high expression.

### The Spearman’s value did not show a correlation between PHF20L1 expression and patient progression-free survival or overall survival

Spearman’s correlation analysis was performed using the percentage of cells expressing PHF20L1 and patient progression-free survival and overall survival. The results did not show any significant correlation (Table 3). However, a slightly negative tendency was observed with CA125 serum levels and tissues with slight overexpression of the protein.

**Table 3:** Spearman’s correlation analysis between PHF20L1 expression determined by IHC and clinical data of the patients

PHF20L1 expression in SKOV-3	Spearman r				
	Age	Clinical stage	CA125	PFS	OS
PHF20L1 total expression 95% CI	0.1336	− 0.2294	− 0.2719	− 0.1517	− 0.0665
Weak Expression 95% CI	0.294	− 0.27	− 0.4793	0.1675	0.33
Moderate Expression 95% CI	− 0.1069	− 0.2104	0.01281	− 0.1527	− 0.299
High Expression 95% CI	− 0.1306	0.007537	0.2027	− 0.1536	− 0.2112

### Impact of PHF20L1 expression on patient survival

**Fig. 3:**
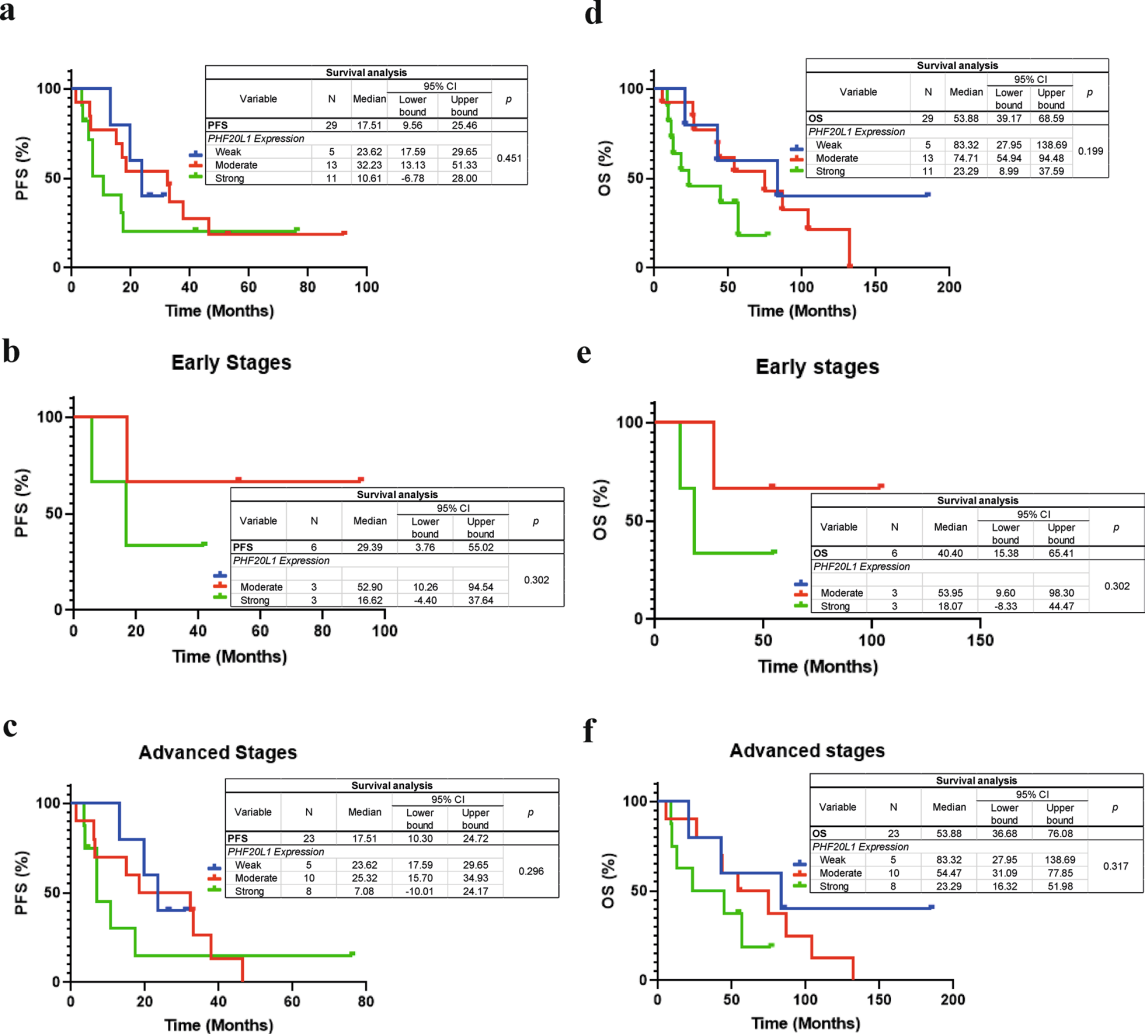
Progression-free survival and overall survival analyses. The data correspond to the survival curve concerning the expression of PHF20L1, which was grouped into weak, moderate, or high expression, based on the immunoreactive score (ISR) value. In the left panel, PFS is reported (**a**–**c**). The right panel indicates the OS (**d**–**f**). From top to bottom: all ovarian cancer patients, only early-stage patients, and only advanced-stage patients

PHF20L1 expression in tumor tissue was determined according to the immunoreactive score (Fig. 3). Progression-free survival (PFS) and overall survival (OS) analyses were performed as a function of the level of PHF20L1 expression. The median PFS was 23.62 months (95% CI 17.59–29.65), 32.23 months (95% CI 13.13–51.33) and 10.61 months (95% CI − 6.78 to 28.00) (*ρ* = 0.451) for the weak, moderate and strong expression groups, respectively (Fig. 3a). The median OS was 83.32 months (95% CI 27.95–138.69), 74.71 months (95% CI 54.94) and 23.29 months (95% CI 8.99–37.59) (*ρ* = 0.199) for the weak, moderate and strong expression groups, respectively (Fig. 3b). High expression levels of PHF20L1 correlated with a lower percentage of PFS and OS even when the samples were stratified into early and advanced stages, but without statistical significance (Fig. 3b–f). These results were more evident and similar to the general data of advanced stage patients (Fig. 3c and f).

PHF20L1 does not have DNA-binding sites, but it does have domains that read modifications in histone and nonhistone-like proteins, which are of great importance in epigenetic regulation [9] [17–20]. Excessive levels of enzymes that act as epigenetic modifiers have been reported as biomarkers of cancer and are involved in tumor progression [21]. We previously demonstrated the overexpression of PHF20L1 in tumor tissue; however, we do not know the functional role of this protein in ovarian cancer cells. We demonstrated that the PHF20L1 protein is overexpressed in tumor tissues of patients with different ovarian cancer histotypes and that the level of expression increases as the disease progresses. Thus, we decided to further study this protein and its possible function in ovarian cancer cells using ovarian cancer-derived cell lines.

### Ovarian cancer ascites induce changes in the expression of PHF20L1 isoforms

Ascites are heterogeneous fluids containing various compounds, including cytokines, growth factors, and extracellular matrix compounds, in addition to cells; thus, ascites are considered a part of the tumor microenvironment. PHF20L1 has multiple isoforms, and only three isoforms are expressed with described molecular weights of 130, 66, and 37 kDa. Given that ascites can modulate protein expression and, thereby, the cellular phenotype in ovarian cancer cells [15], the expression of PHF20L1 isoforms was analyzed to determine whether this ovarian cancer microenvironment could modulate their expression. SKOV-3 cell cultures were treated with ten different ascites fluid samples from epithelial ovarian cancer patients to evaluate their effect on PHF20L1 protein expression in the cell line by taking into account the heterogeneity of ascites.

**Fig. 4:**
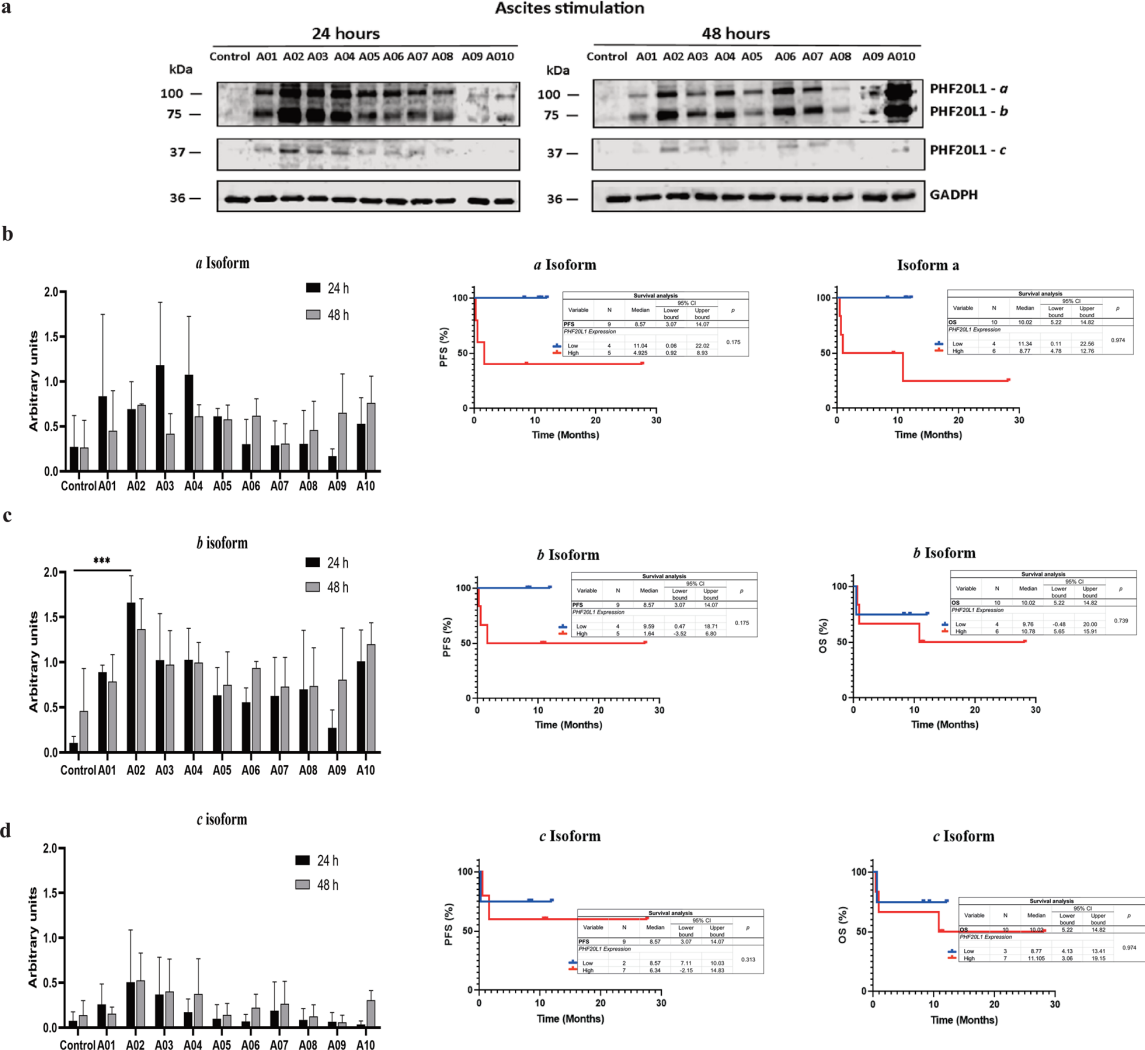
PHF20L1 isoform expression in SKOV-3 cells under stimulation with EOC ascites. Western blot assays were performed in three independent experiments to analyze PHF20L1 expression, and a representative image is shown in **a**. Densitometric analyses, PFS and OS were performed and are shown in **b**, **c** and **d** graphs that correspond to "*a*", "*b*", and "*c*" isoforms, respectively, after 24 or 48 h of stimulation with ascites. For the PFS and OS curves, only 48-h expression values were reported. Statistical analysis was performed using the GraphPad program. Significant differences are shown and correspond to ***ρ < 0.0001. GAPDH was used as a loading control

It was possible to analyze the expression of the three isoforms of the protein that showed molecular weights of 115, 63, and 37 kDa in western blot assays in our experimental conditions. These isoforms were denominated as “*a*”, “*b*”, and “*c*” isoforms, respectively (Fig. 4). Under the control condition, expression was almost undetectable. All isoforms increased their expression after stimulation with the ascitic fluid samples (Fig. 4a). At 24 h, the three isoforms were observed. However, at 48 h, the expression increased in some of the ascites, although the results were not significant. These assays were performed in three independent experiments, and the densitometric analyses of these results were plotted (Fig. 4b–d). Complete images of western blot assay are shown in Additional file 7: Fig. S6.

Moreover, OS and PFS curves were generated based on samples with low and high protein expression. In all cases, the high expression group had a survival rate equal to or less than 50%, whereas the low expression group showed a survival rate of 100% in PFS and higher than 70% in OS. However, no one was statistically significant. A Spearman’s correlation analysis was also performed using the clinical variables obtained from the records of patients with ovarian cancer (from the ten patients described in Table 2). Results are shown in Table 4. The expression of the “*c*” isoform of PHF20L1 showed a trend that could suggest a negative correlation with clinical stage, however, this correlation did not show statistical significance. This analysis was performed also with the data from 24 h incubation and no significant differences were obtained either (data not shown).

**Table 4:** Spearman’s correlation analysis between PHF20L1 expression and patient clinical data

Expression of PHF20L1 in SKOV-3 cells	Spearman r				
	48 h of stimulation with ascites				
	Age	Clinical Stage	CA125	PFS	OS
Isoform *a* 95% CI	0.5547	− 0.08785	− 0.4736	− 0.2940	0.009471
P-value	ns	ns	Ns	ns	ns
	0.0960	0.8093	0.1668	0.4425	0.9793
Isoform *b* 95% CI	0.1425	− 0.2995	− 0.1924	0.05859	0.2580
P-value	ns	ns	Ns	ns	ns
	0.6944	0.4006	0.5943	0.8810	0.4717
Isoform *c* 95% CI	0.01298	− 0.4900	− 0.2969	0.03576	0.2448
P value	ns	ns	Ns	ns	ns
	0.9716	0.1505	0.4048	0.9272	0.4975

### Ascites induce PHF20L1 overexpression also in OVCAR-3 cells

**Fig. 5:**
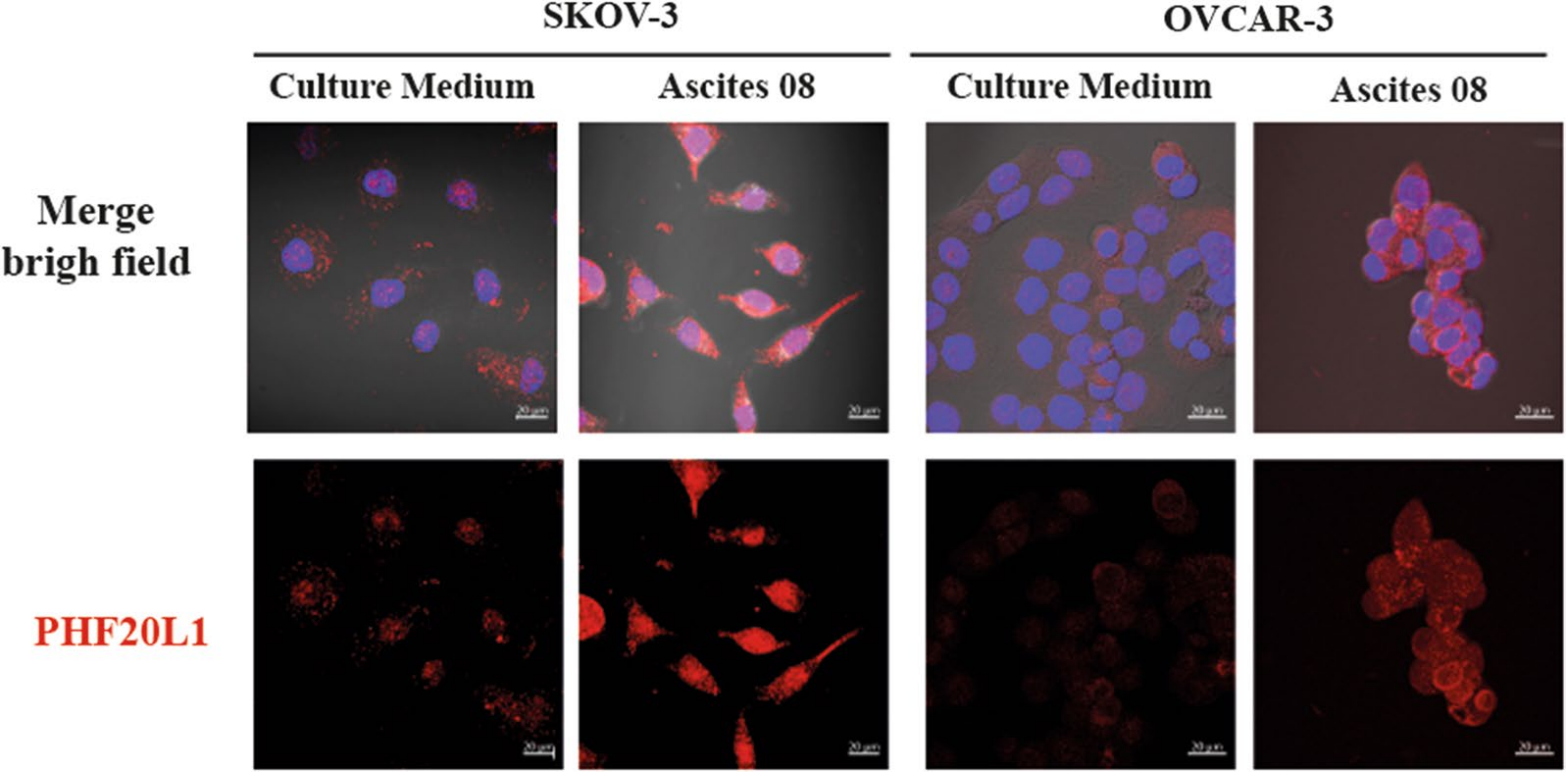
PHF20L1 expression in SKOV-3 and OVCAR-3 cells assessed by immunofluorescence under stimulation with different ascites samples. Immunofluorescence of SKOV-3 and OVCAR-3 cells that were stimulated with different ascitic fluid samples for 24 h. PHF20L1 protein is observed in red (anti-rabbit (IgG) secondary antibody conjugated with Alexa Fluor 647 or anti-rabbit IgG conjugated with TRITC, as corresponding). Nuclei were stained with DAPI and observed in blue

Similarly, using immunofluorescence assays, the expression level and subcellular location of the protein in ovarian cancer cell lines (SKOV-3 and OVCAR-3) were analyzed (Fig. 5). PHF20L1 was localized both in nucleus and in the cell cytoplasm. SKOV-3 cells, after the stimulation with some ascites, showed an increased expression compared with the control condition, as previously shown by western blot assays. In OVCAR-3 cells, because the basal expression level was high, the increase in PHF20L1 expression due to ascites stimulation was not as evident; however, a slight increase was observed compared to nonstimulated cells. To confirm that the induction of PHF20L1 expression by ascites fluid was a generalized phenomenon, the expression of PHF20L1 in SKOV-3 cells was analyzed using nine different ascites samples (Additional file 4: Fig. S4). Five different ascites samples were used in OVCAR-3 cells (Additional file 5: Fig. S5).

## Discussion

EOC is the deadliest gynecological cancer. A prerequisite for reducing the number of deaths involves the availability of adequate diagnostic and monitoring tools that allow physicians to make accurate and timely decisions. In this work, PHF20L1 protein was analyzed because it was previously identified as a fucosylated protein in ovarian cancer cell lines with an aggressive phenotype [10] to evaluate its potential as a key component in the pathogenesis and/or as a possible biomarker of ovarian cancer. Studies have assessed the expression of this protein in breast cancer tissues [9, 12], but no study has assessed PHF20L1 expression in EOC prior to ours [10].

Immunohistochemical detection of PHF20L1 in 29 tumor tissues from different patients with epithelial ovarian cancer showed increased expression of the protein (Fig. 1; Additional file 2: Fig. S2) compared to control tissues (cancer-free tissues) where the expression was very low (the immunoreactive score values were considered negative and low for these two tissues) (H1 and H2, Additional file 6: Table S1). PHF20L1 in the samples in our study was also compared with that reported in the Human Protein Atlas database, which indicated that the expression was confined to the stroma and follicular cells in a healthy ovary [8]. It is important to mention that we found discrete expression in the stroma, but the expression was less evident than that noted in the remainder of the tissue sample. These results strongly suggest an increase in PHF20L1 protein expression in EOC tissue (Additional file 7: Fig. S6).

The interaction of SOX-2 with PHF20L1 has already been demonstrated by others [23]; SOX-2 and PHF8, another PHD finger protein, have been analyzed in ovarian cancer tumor tissues [22], showing that SOX-2 was overexpressed in mucinous carcinoma. In contrast, PHF8 was found to be overexpressed in clear cell carcinoma compared to the other histotypes. Both genes, SOX-2 and PHF8, were also detected in the cells of the epithelial surface of a normal ovary [22]. However, as our results show, the overexpression of PHF20L1 was not associated with any particular histotype.

Our results suggest that patients with higher PHF20L1 expression exhibit a shorter progression-free survival period compared with patients with lower protein expression, where the progression-free survival period was longer. In the OS analysis, we noted a trend for patients with higher protein expression to have a shorter survival time. However, the differences were not significant. This was also the case when we analyzed the isoforms by western blotting, even though no significant differences were found in any of the isoforms. Another aspect to consider is the sample size or that different clinical factors could influence the behavior of the correlation with these survival parameters. Thus, it would be necessary to increase the number of samples representing different clinical stages. We propose that if the sample size is increased, true significance would be observed. Nevertheless, based on our results, we strongly suggest an association between the overexpression of PHF20L1 and poor prognosis in ovarian cancer patients.

Protein isoform expression was analyzed in in vitro assays using SKOV-3 cells stimulated with ascites from patients with EOC (Fig. 4). Ascites from EOC patients exhibited modulation of PHF20L1 isoform expression, both at 24 and 48 h of ascites stimulation. According to previous work in our laboratory, cells stimulated with ascites acquire characteristics of a more aggressive phenotype compared with cells not in contact with this fluid [15], and this finding indirectly supports the association between PHF20L1 and this aggressive phenotype. Regarding the modulation of PHF20L1 expression, in a recently published article, it was suggested that the MYC genes and genes that respond to hypoxia, such as HIF1α, could regulate the expression of PHF20L1 [9]. MYC is a gene frequently involved in cancer [24], and it is one of the four genes (in addition to Oct4, Sox2, and Klf4) that can reprogram fibroblasts to become pluripotent stem cells [25–27]. MYC expression is regulated by the activation of different signaling pathways, including the WNT pathway, through receptor tyrosine kinase and TGF-β [26]. As TGF-β is one of the main components of ascites [28], it is possible to hypothesize that, through MYC regulation, PHF20L1 protein could be overexpressed upon ascites stimulation. However, in tumor tissue, where we also observed high expression of PHF20L1, its regulation could be associated with both TGF-β, which is overexpressed in ovarian cancer tumor tissue [29], and with other components given that signaling via WNT or receptor tyrosine kinase would make more sense [30]. It will be necessary to perform additional experiments under the induction of TGF-β, for example, or with an MYC knockdown animal model to corroborate whether MYC absence would affect the expression of PHF20L1.

On the other hand, ascites fluid stimulates the expression of all the isoforms of PHF20L1 since very little expression is found in medium SKOV-3 cells, all the isoforms were overexpressed at 48 h, and all of them were expressed from 24 h, apart from isoform c, which is almost not expressed some of the ascites (Fig. 4). Studies performed to date by other groups do not highlight any particular isoform’s relevance, except for “c” isoform, which was reported to interact with the DNMT1 protein [20].

However, based on our results, we suggest that PHF20L1 could eventually represent an EOC biomarker. To achieve this goal, it will be necessary to increase the number and clinical stages of EOC samples to analyze the expression level of PHF20L1; furthermore, correlation analyses with the expression of additional markers, such as CA125 or Ki67, in tumor tissues should be performed given that these molecules may indicate recurrence or response to therapy (CA125) or a high rate of cell proliferation (Ki67).

## Conclusion

In conclusion, even though this work presents certain limitations to conclusively affirm that high PHF20L1 expression is associated with lower progression free survival and overall survival, our data constitute the first approximation to determine the expression of PHF20L1 in ovarian cancer tissue and cell lines stimulated by malignant ascites. Based on the results, we suggest that this protein could play an important role in these tumor cells. However, a more detailed analysis will be necessary to corroborate the specific role of PHF20L1.

## Supplementary Information

**Additional file 1: Figure S1.** Secondary antibody controls used in the immunohistochemistry technique.

**Additional file 2: Figure S2.** Representative images of the 31 sections of tumor tissue analyzed by IHC. A representative image per slice is attached, corresponding to 10X and 20X magnification for each tissue analyzed. The tissues were numbered from 1 to 29, which correspond to tumor tissues from patients with ovarian cancer. A code of numbers was placed in the upper left part, which represents the sample number, clinical stage, and histological subtype in order from left to right. The numbering corresponds to the following: clinical stage: 1: IA, 3: IC, 6: IIIA, 7: IIIB, 8: IIIC, 9: IVA, 10: IVB; histological subtype: 1: HGSP, 2: LGSP, 3: endometrioid, 4: CC, 5: mucinous.

**Additional file 3: Figure S3.** PHF20L1 expression in the cytoplasm or nucleus in sections of tumor tissue with EOC. **a,** shows an image and an enlargement (inset) where expression of PHF20L1 protein is highlighted in the cytoplasm and nucleus. **b,** shows an image and the enlargement of a zone (inset) where the protein expression is located mainly at the nuclear level.

**Additional file 4: Figure S4.** PHF20L1 expression by immunofluorescence in SKOV-3 cells under the stimulus of nine different ascites samples. Cells were incubated with ascites for 24 and 48 h and then analyzed. PHF20L1 protein appears in red (anti-rabbit (IgG) secondary antibody conjugated with Alexa Fluor 647). Nuclei were stained with DAPI and appears in blue.

**Additional file 5: Figure S5.** PHF20L1 expression in OVCAR-3 cells under stimulation with five different ascites. Immunofluorescence of OVCAR-3 cells that were stimulated with different ascitic fluids for 24 h. PHF20L1 protein appears in red (anti-rabbit IgG conjugated with TRITC). Nuclei were stained with DAPI and appear in blue.

**Additional file 6: Table S1.** General data of the patients whose tissues were used in this study.

**Additional file 7: Figure S6.** Original images of Western blot analysis of PHF20L1 stimulation by different ascites at 24 and 48 h.

## Data Availability

Source data of this study were derived from the public repositories, as indicated in the section of “Materials and methods” of the manuscript and all data that support the findings of this study are available from the corresponding author upon reasonable request.

## Abbreviations

EOC: Epithelial ovarian cancer
PHF20L1: PHD finger protein 20-like 1
