# Supplementary figures and images for: PHD finger protein 20-like protein 1 (PHF20L1) in ovarian cancer: from its overexpression in tissue to its upregulation by the ascites microenvironment
Source: Cancer Cell Int. 2022 Jan 6;22:6. doi: 10.1186/s12935-021-02425-6 (PMC8740351; doi:10.1186/s12935-021-02425-6)

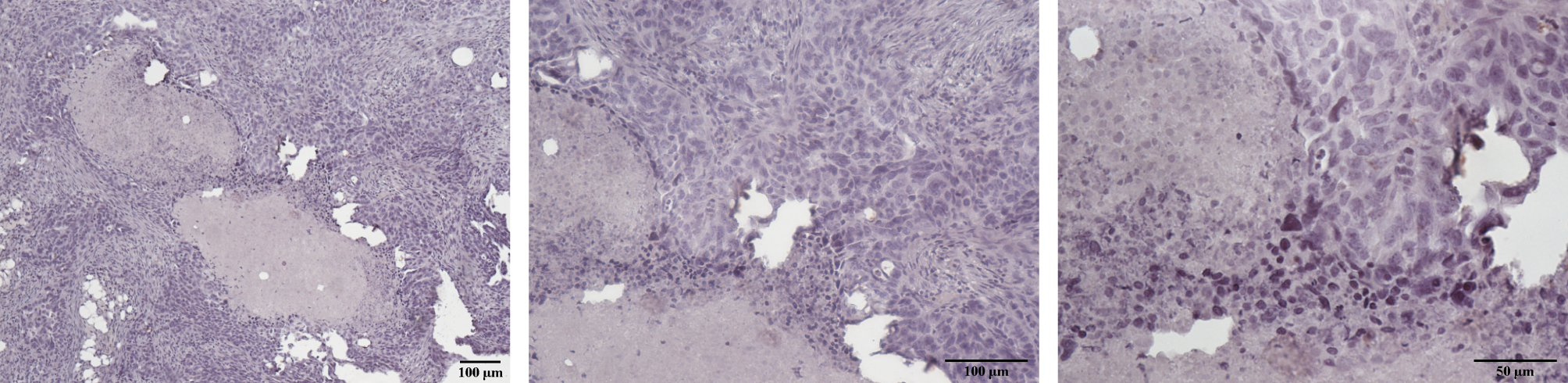

Supplement: Supplementary file 1 — Additional file 1: Figure S1. Secondary antibody controls used in the immunohistochemistry technique. [file 12935_2021_2425_MOESM1_ESM.tif]

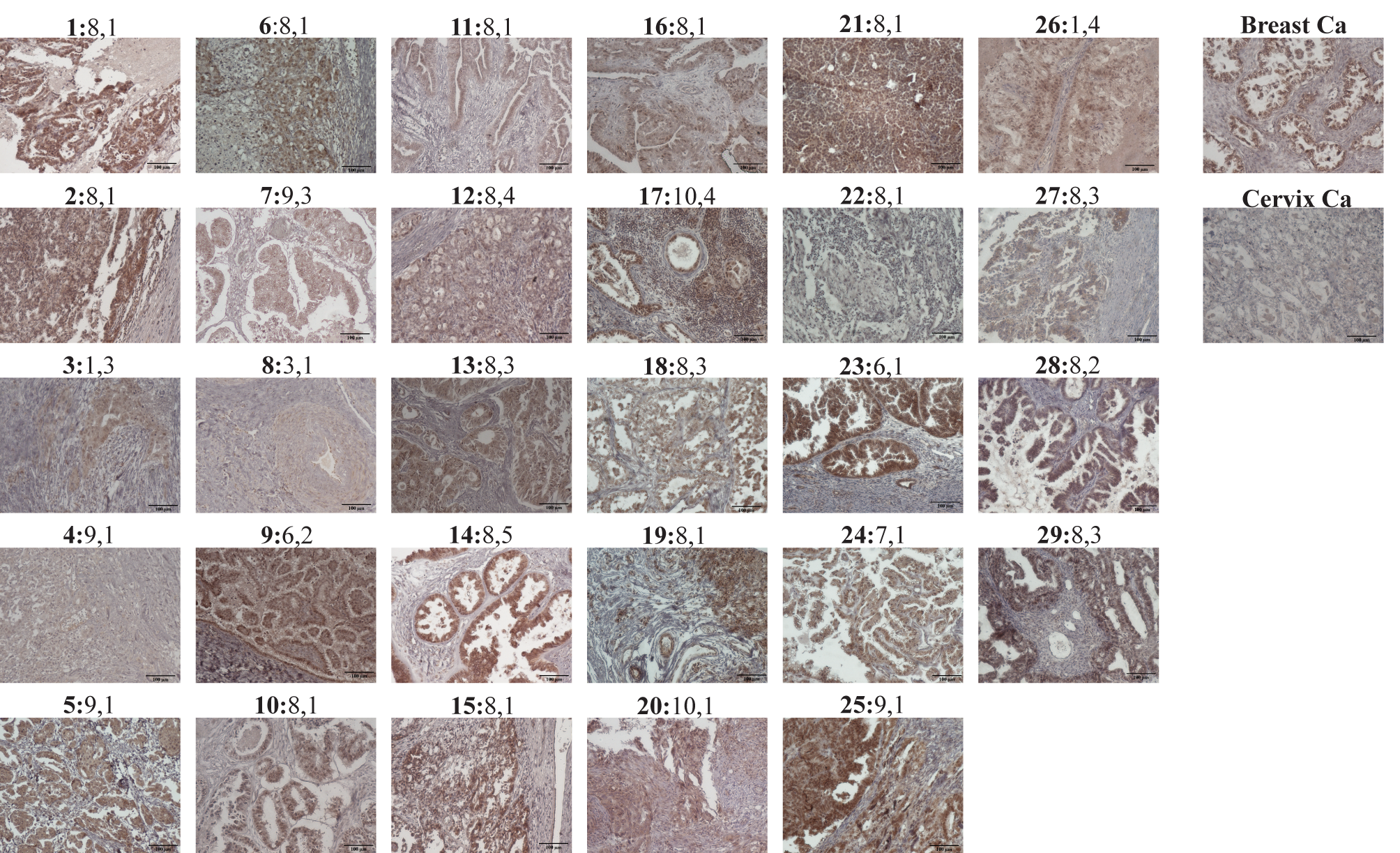

Supplement: Supplementary file 2 — Additional file 2: Figure S2. Representative images of the 31 sections of tumor tissue analyzed by IHC. A representative image per slice is attached, corresponding to 10X and 20X magnification for each tissue analyzed. The tissues were numbered from 1 to 29, which correspond to tumor tissues from patients with ovarian cancer. A code of numbers was placed in the upper left part, which represents the sample number, clinical stage, and histological subtype in order from left to right. The numbering corresponds to the following: clinical stage: 1: IA, 3: IC, 6: IIIA, 7: IIIB, 8: IIIC, 9: IVA, 10: IVB; histological subtype: 1: HGSP, 2: LGSP, 3: endometrioid, 4: CC, 5: mucinous. [file 12935_2021_2425_MOESM2_ESM.tif]

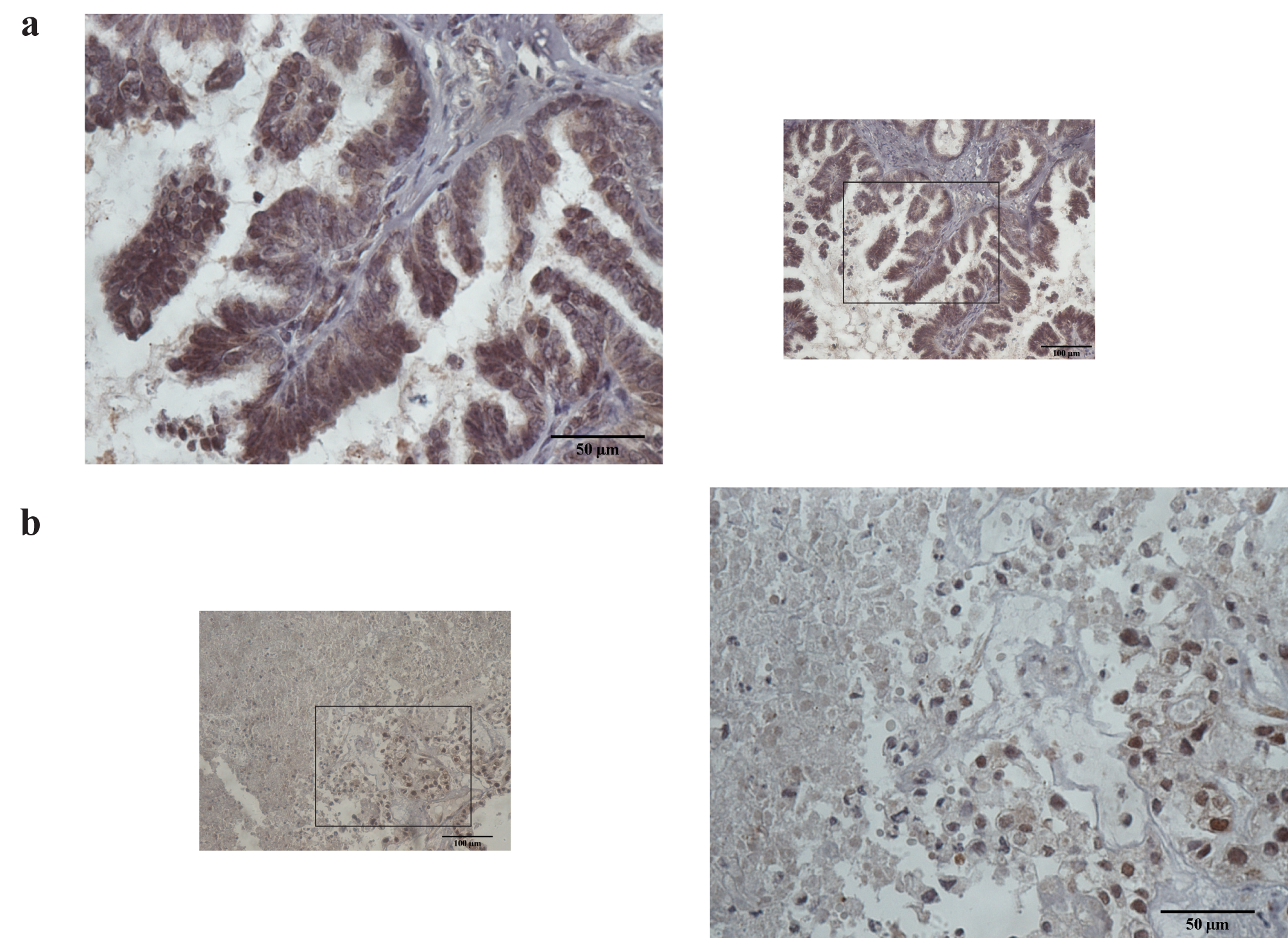

Supplement: Supplementary file 3 — Additional file 3: Figure S3. PHF20L1 expression in the cytoplasm or nucleus in sections of tumor tissue with EOC. a, shows an image and an enlargement (inset) where expression of PHF20L1 protein is highlighted in the cytoplasm and nucleus. b, shows an image and the enlargement of a zone (inset) where the protein expression is located mainly at the nuclear level. [file 12935_2021_2425_MOESM3_ESM.tif]

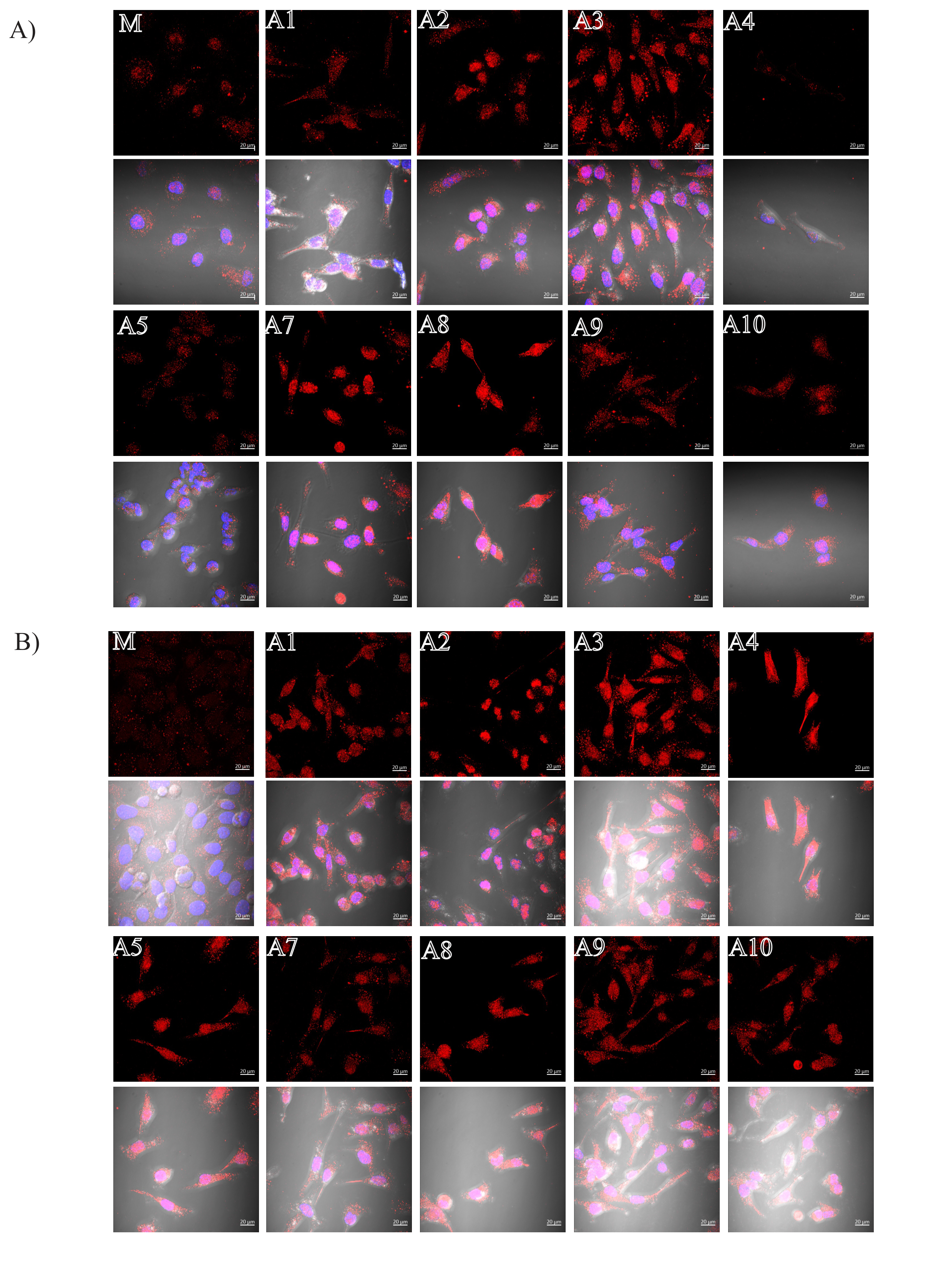

Supplement: Supplementary file 4 — Additional file 4: Figure S4. PHF20L1 expression by immunofluorescence in SKOV-3 cells under the stimulus of nine different ascites samples. Cells were incubated with ascites for 24 and 48 h and then analyzed. PHF20L1 protein appears in red (anti-rabbit (IgG) secondary antibody conjugated with Alexa Fluor 647). Nuclei were stained with DAPI and appears in blue. [file 12935_2021_2425_MOESM4_ESM.tiff]

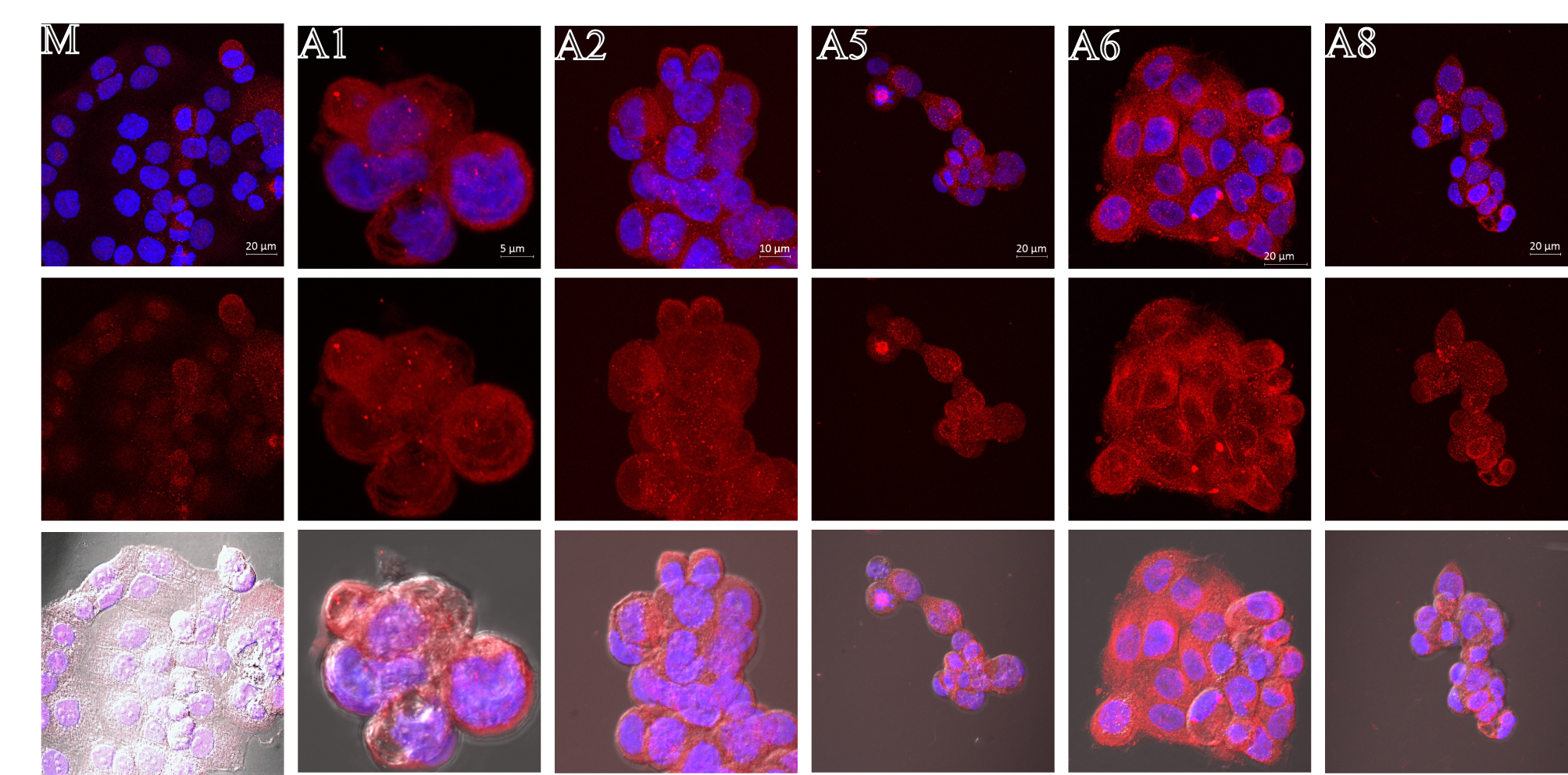

Supplement: Supplementary file 5 — Additional file 5: Figure S5. PHF20L1 expression in OVCAR-3 cells under stimulation with five different ascites. Immunofluorescence of OVCAR-3 cells that were stimulated with different ascitic fluids for 24 h. PHF20L1 protein appears in red (anti-rabbit IgG conjugated with TRITC). Nuclei were stained with DAPI and appear in blue. [file 12935_2021_2425_MOESM5_ESM.tif]

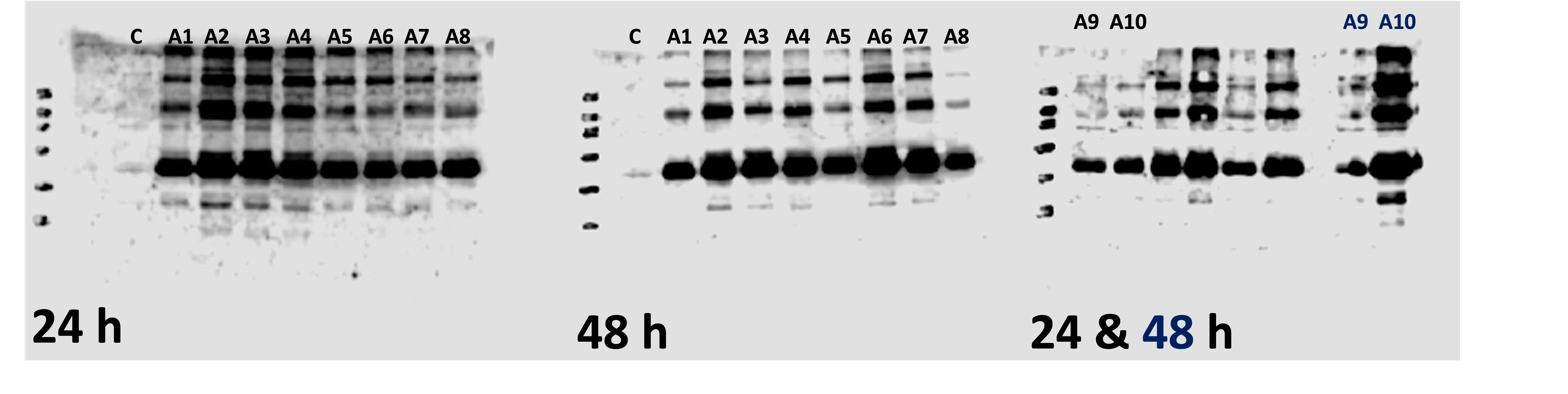

Supplement: Supplementary file 7 — Additional file 7: Figure S6. Original images of Western blot analysis of PHF20L1 stimulation by different ascites at 24 and 48 h. [file 12935_2021_2425_MOESM7_ESM.jpg]
